# Recent advances of three-dimensional bioprinting technology in hepato-pancreato-biliary cancer models

**DOI:** 10.3389/fonc.2023.1143600

**Published:** 2023-04-28

**Authors:** Xiaomei Zhuang, Gang Deng, Xiaoying Wu, Juping Xie, Dong Li, Songlin Peng, Di Tang, Guoying Zhou

**Affiliations:** ^1^Scientific Research Center, The Seventh Affiliated Hospital, Sun Yat-sen University, Shenzhen, China; ^2^Department of General Surgery, The Seventh Affiliated Hospital, Sun Yat-sen University, Shenzhen, China

**Keywords:** 3D bioprinting, bioink, pancreatic cancer, colorectal cancer, hepatocellular carcinoma, cholangiocarcinoma, tumor immune microenvironment, tumor model

## Abstract

Hepato-pancreato-biliary (HPB) cancer is a serious category of cancer including tumors originating in the liver, pancreas, gallbladder and biliary ducts. It is limited by two-dimensional (2D) cell culture models for studying its complicated tumor microenvironment including diverse contents and dynamic nature. Recently developed three-dimensional (3D) bioprinting is a state-of-the-art technology for fabrication of biological constructs through layer-by-layer deposition of bioinks in a spatially defined manner, which is computer-aided and designed to generate viable 3D constructs. 3D bioprinting has the potential to more closely recapitulate the tumor microenvironment, dynamic and complex cell-cell and cell-matrix interactions compared to the current methods, which benefits from its precise definition of positioning of various cell types and perfusing network in a high-throughput manner. In this review, we introduce and compare multiple types of 3D bioprinting methodologies for HPB cancer and other digestive tumors. We discuss the progress and application of 3D bioprinting in HPB and gastrointestinal cancers, focusing on tumor model manufacturing. We also highlight the current challenges regarding clinical translation of 3D bioprinting and bioinks in the field of digestive tumor research. Finally, we suggest valuable perspectives for this advanced technology, including combination of 3D bioprinting with microfluidics and application of 3D bioprinting in the field of tumor immunology.

## 1 Introduction

Hepato-pancreato-biliary (HPB) cancer is a serious category of cancer developing in digestive system, including liver, pancreas, intra- and extra-hepatic biliary ducts (1). HPB cancer is often diagnosed at the advanced stage and associated with aggressive progression and extremely poor prognosis (2, 3). Specially, primary liver cancer is the sixth most frequently diagnosed cancer (4.7% of total cancer cases) and the third leading cause of cancer death (8.3% of total cancer deaths) globally in 2020, with approximately 906,000 newly diagnosed cases and 830,000 deaths (3). The incidence of pancreatic cancer has doubled over the past three decades, increasing from 196,000 newly diagnosed patients in 1990 to 495,773 in 2020 (3, 4). Moreover, pancreatic cancer is the seventh leading cause of cancer death, accounting for almost as many annual deaths (466,003) as new cases (495,773) (3). Gallbladder carcinoma (GBC), ranks as the sixth most frequently diagnosed gastrointestinal malignancy also with poor prognosis (5, 6). Unfortunately, only a small population of GBC patients (10%-30%) is eligible for surgical resection which is the current effective treatment (7). HPB cancer is complicated, and its diagnosis and treatment are diverse and interdisciplinary (8). Although remarkable progress has been made in fundamental research on HPB cancer, with respect to tumor microenvironment, immunotherapy, organ transplantation, and so on (9–13), it is still not completely clear in mechanisms of cancer development and there is a lack of curative treatments. Therefore, it poses an urgent need for better understanding of tumorigenesis in HPB cancer and for improved therapeutic strategies.

In oncology research, conventional two dimensional (2D) monolayer cell culture and animal models have been widely used to mimic the *in vivo* milieu and predict pathophysiological and toxicological responses to drugs, but evidence has shown that they cannot accurately predict clinical response or reflect cellular microenvironment, which might lead to false-positive or false-negative drug selection, inefficient prediction of drug response and economic loss (14). Currently, it is urgent to develop experimental system with an inherent ability to recapitulate the complex tumor biology. Therefore, novel three dimensional (3D) culturing technologies, including spheroid and organoid cultures, are developed to contribute to reproducing intercellular communication, intricate architecture of the tumor microenvironment and contact with the extracellular matrix (ECM) (14). It has been proved that the biological and predictive superiority of 3D systems is over conventional 2D culture methods (15, 16). The next level for developing more accurate and dynamic tumor models for oncology research and personalized medicine is advancing to new biological technologies like 3D bioprinting.

3D bioprinting is a reproducible bio-fabrication technology capable of generating biological constructs similar to their native counterparts with high spatial precision and controllability (17–20). It precisely positions biologics including heterogeneous cells, biological materials, biochemicals and other biological entities by an automated dispensing system (20, 21). The quality of the bioprinted scaffold can be influenced by the extracellular microenvironment, cellular response, biocompatibility and biodegradability (22). By virtue of its potential in tissue and organ regenerative engineering, drug screening, patient-specific therapies, organ-on-a-chip and high-throughput screening, 3D bioprinting has gained increasing attention.

The process of 3D bioprinting refers to printing and patterning bio-functional materials in a manner of layer-by-layer on substrates or culture dishes containing cell culture medium to maintain cellular adhesion and sustained growth (18, 20, 22). Bio-functional materials include living cells, basic structure materials and other essential components, which are defined as bioinks (23, 24). To reproduce the complex and heterogeneous architecture of organs and tissue, gaining a comprehensive and sufficient understanding of composition and organization of their components is essential. For this purpose, medical imaging technology, as an indispensable tool, can provide the information on 3D structure and function at cellular, tissue, organ and organism levels. These technologies include common and noninvasive imaging modalities like magnetic resonance imaging (MRI) and computed tomography (CT) (20). Computer-aided design and computer-aided manufacturing (CAD-CAM) tools, mathematical modeling and machine learning are also used to collect and digitize the complex tomographic and architectural information for tissue (24–26).

In this review, we will introduce and compare multiple types of 3D bioprinting methodologies and commonly used biomaterials for HPB cancer and other digestive tumors. We will then discuss the advances and applications of 3D bioprinting in HPB cancer research and other gastrointestinal cancers, focusing on tumor model manufacturing. We will also highlight the current challenges regarding clinical translation of 3D bioprinting and bioinks in the field of digestive tumor research and suggest valuable perspectives for this advanced technology.

## 2 3D bioprinting methodologies for HPB cancer and other digestive tumors

The success of 3D culturing model or tissue engineering chiefly depends on the capability to formulate complicated cell-laden 3D structures that mimic native tissue or organs. Therefore, methodologies used for designing and creating the architecture are an important aspect of 3D bioprinting. In the field of HPB and other digestive tumor research, there are different bioprinting methodologies depending on fundamental working principles (27), including extrusion-based bioprinting, laser-assisted bioprinting, inkjet-based bioprinting, magnetic bioprinting, coaxial bioprinting and acoustic bioprinting (**Table 1**).

**Table 1:** Features and applications of different strategies of 3D bioprinting.

3D bioprinting strategy	Advantages	Disadvantages	Resolution	Cell density	Clogging possibility	Applications
Extrusion-based bioprinting	-Applicability of multi-material bioprinting -Suitable for bioinks with various viscosities -Printability of high cell density -Simple bioprinting process	-Low printing speed -Relatively low resolution -Relatively low cell viability	Low to medium dependent on setup of bioprinter	High	Yes	-Hepatocellular carcinoma model (28–30) -Colorectal cancer model (14) -Hepatocellular carcinoma model with microfluidic chip (30) -Blood vessels (31, 32)
Laser-assisted bioprinting	-High cell viability -Suitable for bioinks with various viscosities	-Relatively low cell density -Complex setup and system -High cost	High (<500 nm)	Low	No	-Pancreatic cell network (33) -Glioblastoma tumor model (34) -Epithelium-mimicking structures (35) -Voluminous bone tissue (36)
Inkjet-based bioprinting	-Low cost -High cell viability -High resolution -Simple bioprinting process	-Limited by the viscosity of bioink -Low cell density -Frequent clogging of nozzles -Unequal sized drops	High (>50 μm)	Low	Yes	-Microvascular tissue (37) -Mille-Feuille-like 3D structure (38) -Skin (39) -Blood vessels (40)
Magnetic bioprinting	-Print in a high-throughput way -Low cost	-Limited by bioink materials	High	High	No	-Prostate tumor (41) -Adipose tissue (42) -Lung (43) -Aortic valve (44)
Coaxial bioprinting	-Printability of tissue with built-in microchannels or heterogeneous properties	-Low printing speed -Relatively low cell viability	Low to medium dependent on bioprinter	High	Yes	-Microvascular tissue (45) -Liver sinusoid (46)
Acoustic bioprinting	-Printability of high-density cells or single cell -High resolution -High cell viability	-Limited by bioink materials	High (5-300 μm) (47)	High	No	-Colorectal cancer-cancer-associated fibroblasts model (48)

### 2.1 Extrusion-based bioprinting

**Figure 1:**
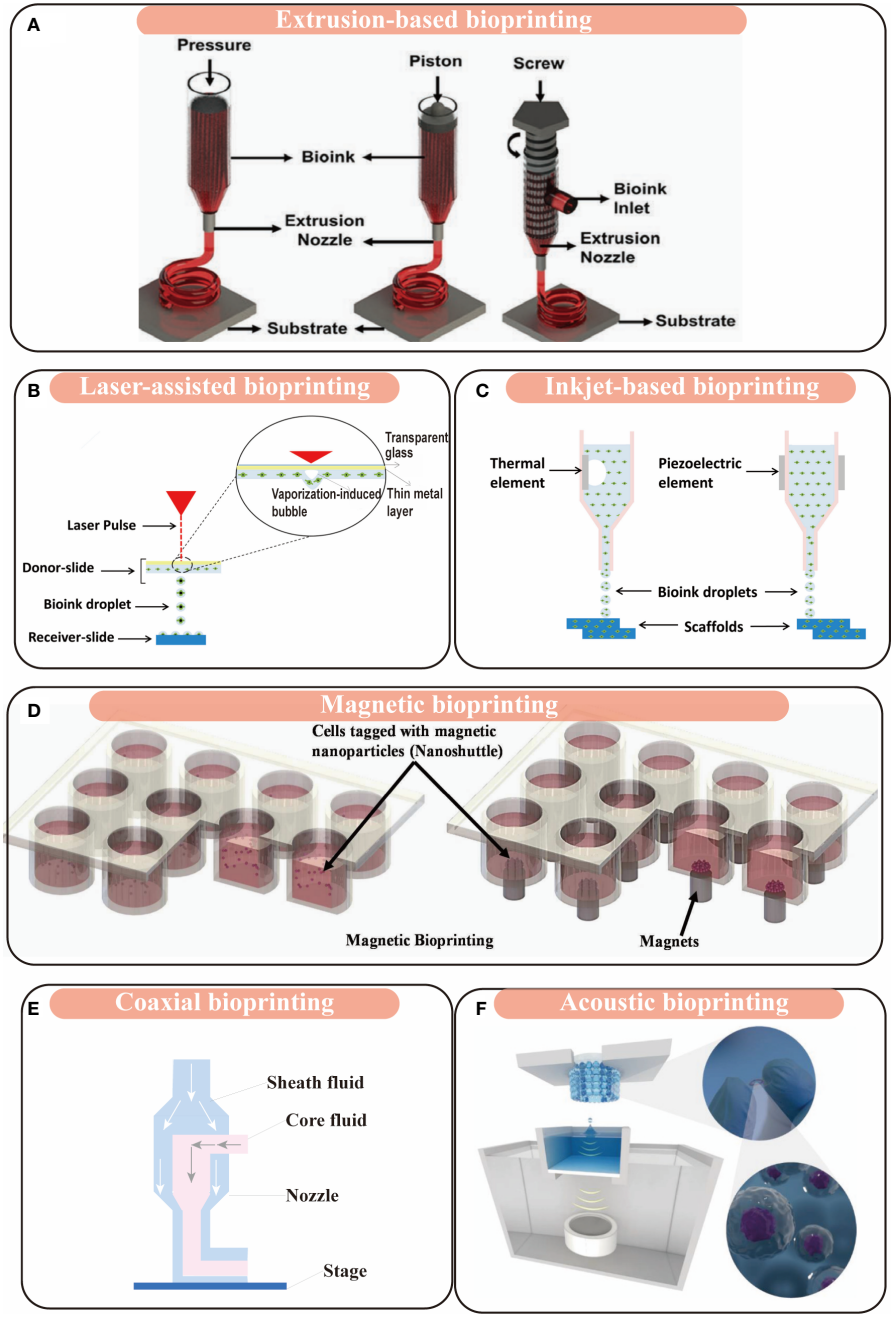
Schematic figures of 3D bioprinting modalities. **(A)** Extrusion-based bioprinting; adapted with permission (18), copyright 2019 John Wiley and Sons. **(B)** Laser-assisted bioprinting; adapted with permission (49), copyright 2018, MDPI (open access). **(C)** Inkjet-based bioprinting; adapted with permission (49), copyright 2018, MDPI (open access). **(D)** Magnetic bioprinting; adapted with permission (21), copyright 2020 ELSEVIER. **(E)** Coaxial bioprinting. **(F)** Acoustic bioprinting; adapted with permission (47), copyright 2021 John Wiley and Sons (open access).

Extrusion-based bioprinting is one of the most popular and versatile 3D bioprinting methodologies and convenient to dispense various bioinks like hydrogels. In extrusion-based bioprinting, the bioink is extruded through a nozzle and dispensed onto substrates pneumatically or mechanically. The bioink (the cell and biomaterial mixture) is drawn into a sterilized syringe with a needle and then the 3D tissue or organ model is fabricated by extrusion in a layer-by-layer manner under control of an automated three-axis robotic system (**Figure 1A**) (21, 27, 28, 50). Materials with various viscosities are compatible with extrusion-based bioprinting because of its pneumatic driven system (51, 52). Even the complex biological tissue can also be achieved by extrusion-based bioprinting using multiple bioinks and setting up multiple printing headings (50, 53). In virtue of the advantages of extrusion-based bioprinting in economy, efficiency, and uniform cell diameter, it has been applied to fabricate various HPB and other digestive tissue and organs, including 3D bioprinted model derived from HepG2 cells (3DP-HepG2), 3D patient-derived bioprinted model for hepatocellular carcinoma (3DP-HCC), 3D bioprinted model with microfluidic chip (3DPF), 3D bioprinted colorectal cancer (CRC) model, etc. (14, 28–30).

### 2.2 Laser-assisted bioprinting

Laser-assisted bioprinting was developed from the initial research of the U.S. Naval Research Laboratory (54). It is an orifice-free, non-contact methodology that can rapidly deposits bioinks onto substrates with spatial accuracy of >5μm (54). This technique is based on the laser-induced forward transfer effect (LIFT). It consists of three main components: 1) an energized pulsed laser; 2) a target or ribbon (including a transparent glass slide coated with a layer of laser-absorbing gold or titanium, onto which bioink is spread), which is the donor film of biological material; and 3) a receiving substrate which collects the printed material (55). It usually allows a laser beam which is in the infrared spectrum to precisely focus on the ribbon, and then the bioink is deposited over the substrate (**Figure 1B**). The main advantages of this technique are that it can deposit bioink with relatively high resolution, viscosity and cell viability (56, 57). Because of its nozzle-free process, it allows for an excellent performance on the cell viability compared with other bioprinting methods, which has been proved to relate to the energy of laser pulse, the substrate thickness of ECM, and the viscosity of bioinks (57). For its ultimate printing precision, it provides with an ultimate control over cell organization and deposition (55). Until now, a group of researches has used this technology to create tissue and organs to mimic their counterparts, such as rat exocrine pancreatic cell network (55), glioblastoma tumor model (34), epithelium-mimicking structure (35), and voluminous bone tissue (36).

### 2.3 Inkjet-based bioprinting

Inkjet-based bioprinting is the most widely known bioprinting technology originally from the 2D desktop inkjet printers. It is a droplet-based bioprinting, offering an efficient and simple method for precise deposition of multiple cells and other various bioink components, especially under the control of drop-on-demand at a level of picoliter (**Figure 1C**) (38, 58, 59). This bioprinting technology is formed by two strategies: drop-on-demand and continuous inkjet printing. As the name suggests, drop-on-demand printing generates bioink drops only when required, while continuous inkjet printing relies on the flow tendency of liquid stream. Unfortunately, inkjet-based bioprinting has several limitations that hinder its application in 3D fabrication. Firstly, the frequent clogging of nozzles and cell sedimentation inside the printhead chamber could impair the precision control of the droplet formation and disturb the smooth printing process (31). Secondly, continuous inkjet printing requires conductive fluid inks, which leads to a challenge in selection of biomaterials. Thirdly, several reports suggest that the thermal and sheer stress in the process of bioink drop formation may affect cell viability (60, 61). To overcome these limitations, several researches made progress in newly designed printhead (38, 62) and cell-laden bioink circulation (63). Takagi et al. reported that they used inkjet-based bioprinter with newly designed printhead to construct a Mille-Feuille-like 3D structure with living NIH/3T3 mouse fibroblast cells and normal human dermal fibroblasts (38). Solis et al. proved that thermal inkjet bioprinting can trigger the activation of the vascular endothelial growth factor (VEGF) pathway in human microvascular endothelial cells, which may offer a new strategy for vascularization in tissue engineered structures (37).

### 2.4 Magnetic bioprinting

**Figure 2:**
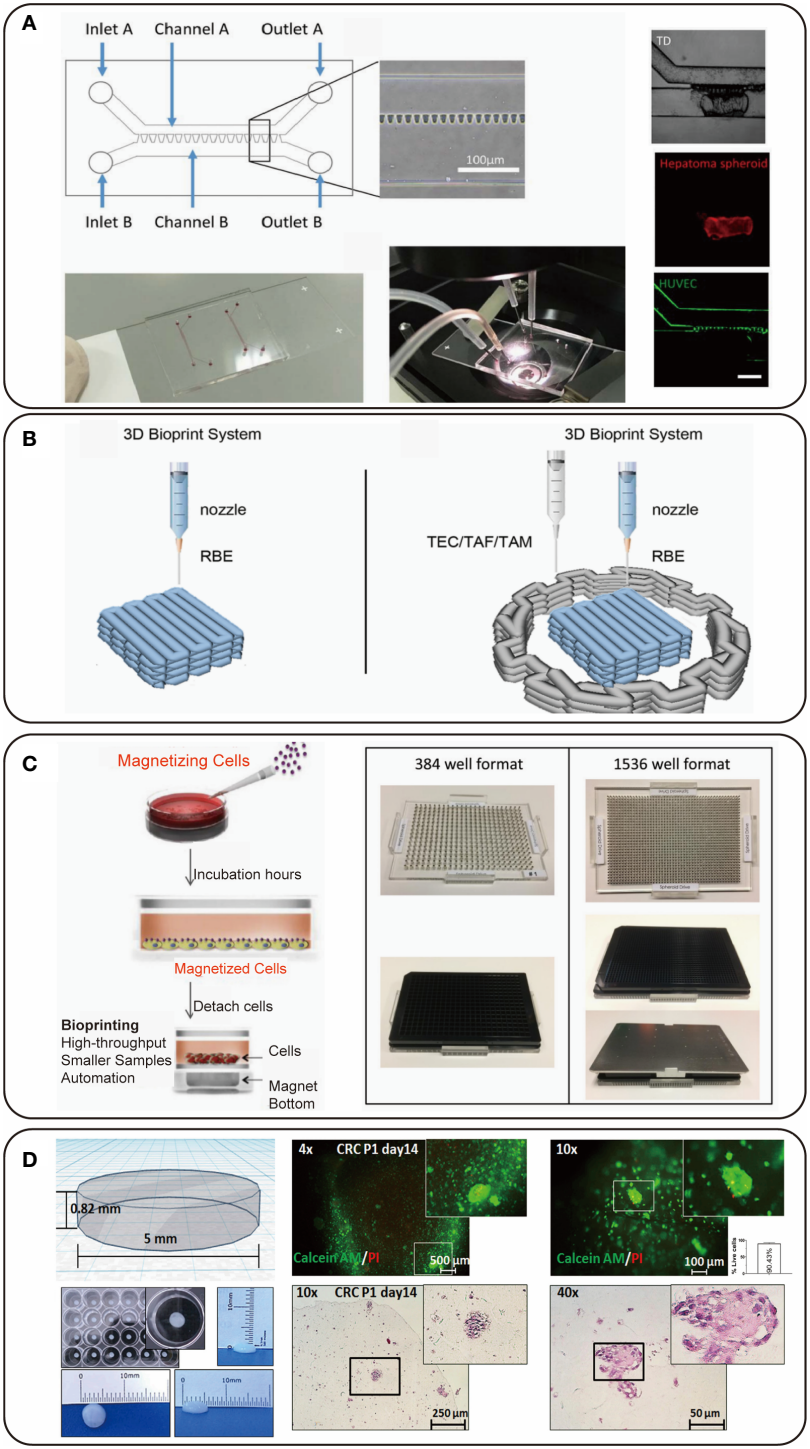
Representative figures of 3D bioprinted hepato-pancreato-biliary (HPB) cancer models. **(A)** The design of a 3D bioprinted hepatocellular carcinoma (HCC) model combined with microfluidic technology; adapted with permission (30), copyright 2019 IOP Publishing. **(B)** A 3D bioprinted model with cholangiocarcinoma (CCA) cells and stromal cells; adapted with permission (65), copyright 2022 Frontiers. **(C)** A 3D bioprinted pancreatic cancer model for high-throughput screening (HTS); adapted with permission (66), copyright 2022 ELSEVIER (open access). **(D)** A 3D bioprinted colorectal cancer model derived from patient; adapted with permission (14), copyright 2021 Frontiers.

In 2013, Haisler et al. described a method for 3D culture based on magnetic levitation, in which a magnetic nanoparticle bound to the cells to render the cells magnetic (64). When the magnetic cells were resuspended in medium, they could be levitated and concentrated at the air-liquid interface by an external magnetic field. In this process, the cells aggregated to form 3D cultures (**Figure 1D**) (64). This method offered the basis for magnetic 3D bioprinting. This technique can synthesize endogenous ECM and provide fine spatial control without needing other artificial protein substrates. Additionally, it can print multiple tissue-like structures in a high-throughput pattern (43, 44). Similar technology has been applied in tissue engineering, such as for prostate tumor (41), adipose tissue (42), lung (43), aortic valve (44), and so forth. The main advantage of magnetic bioprinting is its capability to fabricate native-like tissue in a high-throughput way. Fernandez-Vega et al. applied this method to the first large-scale screening work and completed high-throughput screening (HTS) on over 150,000 small molecular drugs against primary pancreatic cells (**Figure 2C**). They identified four chemotypes of drugs (SR-963, SR-742, SR-667 and SR-444) as leads in 3D pancreatic cancer models, which demonstrates that 3D magnetic bioprinting technology is a powerful tool for fast, affordable and automated HTS (66).

### 2.5 Coaxial bioprinting

Coaxial bioprinting is a relatively new bioprinting method *via* using a coaxial nozzle (31). As the sheath and core channels are fed with biomaterials with different physico-chemical characteristics, this method is especially suitable for fabrication of tissue with built-in microchannels or vertical structures with heterogeneous properties (**Figure 1E**) (45, 67). When we adjust mechanical properties like toughness, shear force, viscosity and compression by selecting appropriate biomaterials, we can use coaxial bioprinting to fabricate various tissue. Alginate and gelatin methacryloyl (GelMA)/gelatin are appropriate materials for bioprinting of vascularized structure (31, 68–70). Bioprinting method based on hollow alginate filaments for vascularized structure allows concurrent printing of scaffold and microchannels. Alginate flow and calcium chloride flow fade sheath and core channel respectively, and the crosslinking of alginate and calcium ion is controlled by crosslinking sequence time, concentration and flow rate of alginate and calcium chloride solutions. GelMA and gelatin solutions are pumped into sheath and core channels of the coaxial nozzle respectively, and gelatin can be as sacrificial materials when temperature rises to obtain microchannels. Based on these features, coaxial bioprinting is suitable for tissue with abundant blood supply such as liver, and liver sinusoidal models developed by coaxial bioprinting have been reported (46). Using customized coaxial nozzle allowed researchers to deposit bioinks with different types of cells sequentially, which allowed formation of vertical structures composed of homogeneous or heterogeneous properties such as intestinal villus and hair follicle (45).

### 2.6 Acoustic bioprinting

Acoustic bioprinting is a novel method based on the principle of acoustic droplet ejection. The acoustic printer adopts an open cartridge and the focus of the ultrasonic signal is close to air-liquid interface. The acoustic streaming effect causes a flow against surface and the formation of a dome on the liquid surface. An amount of liquid is ejected when the sound pressure of ultrasonic signal and its resulting kinetic flow energy overcome the surface tension of the liquid. The liquid droplets are collected by the substrate (**Figure 1F**) (47, 48). The absence of a nozzle allows the printer to minimize cell damage and enables to print single cell, high-density cells and even cell spheroids flexibly. In terms of high resolution, acoustic bioprinting has the advantage of convenient and flexible positioning of tumor cells, normal cells, fibroblasts and extra- cellular matrix, which leads to reproduction of the tumor microenvironment to a large extent. The printed cells maintain high viability (>94%) (48), which is superior to extrusion-based (40%-80%) (58) and inkjet-based (>85%) (71) bioprinting and indicates that acoustic bioprinting has the ability to construct long-term tumor model for simulating dynamic drug response and tumor invasion.

## 3 Selection of bioinks

The main consideration of bioink selection is based on the printability and the impact of bioink on cell behavior. Printability is generally associated with the rheological properties of the bioink. The rheological properties permit extrusion during printing and allow stabilization after deposition onto a substrate. Shear-thinning hydrogels are usually considered as ideal bioinks as they can flow during extrusion and can protect cells from shear stress. Rheological additives, including gelatin and methylcellulose, can be used for inducing shear-thinning behavior (72). The control of polymer concentration can regulate shear-thinning behavior and higher polymer concentrations usually possess improved rheological properties (73). Each bioink presents different biological and mechanical characteristics, bioink selection should also depend on the biological question and the specific cell type. Biological properties such as adhesion to cells and degradation ability are important features to consider in an experiment and may affect encapsulated cell response. Mechanical properties, like elastic modulus, can be translated to biochemical signals and impact cellular responses including proliferation and differentiation (74). However, the controversies between mechanical properties in bioink and cellular viability during bioprinting cause limitations on application. Mechanical and rheological properties in bioink generate tissue constructs with adequate mechanical strength, high shape fidelity, and robustness. However, better printability of bioink usually causes lower cellular viability during cell bioprinting.

Hydrogels including alginate, GelMA, collagen, fibrin, Matrigel, gelatin and polyethylene glycol (PEG) are frequently used in 3D bioprinted HPB tumor models. Among them, alginate is a polysaccharide molecule, consisting of alternating α-l-guluronate and β-d-mannuronate units (75). Alginate undergoes ionic crosslinking *via* the negatively charged carboxylate (COO−) group existing in polymeric backbone. When the COO− group contacts with positively charged ions like divalent calcium cations, a crosslinked hydrogel network is developed (58, 76). This crosslinking mechanism of alginate with CaCl_2_ is exploited to fabricate heterogeneous tissue constructs such as 3D bioprinted hepatocellular carcinoma (HCC) cell model (28) and liver sinusoid models (46). GelMA is a denatured collagen protein, in which methacrylate groups are conjugated to amine side groups (77). GelMA has been increasingly used for 3D bioprinting benefiting from its controllable mechanical characteristics and appealing biocompatibility. Compared with polymers derived from natural sources, GelMA forms an enzymatically degradable hydrogel with higher mechanical strength when photocrosslinked with ultraviolet light (78). Collagen is a thermosensitive hydrogel and a major component of ECM in HPB tissue. In virtue of its biocompatibility and cell adhesive properties, it has been extensively exploited in 3D bioprinting for HPB and other tissue (79), including hepatic lobule-like structure (52). Fibrin is a hydrogel formed *via* the reaction of fibrinogen with thrombin, supporting cell growth and proliferation (61).

In addition, the dECM derived from nature’s own scaffold has become a new bioink source (80). The dECM scaffolds of 3D bioprinted liver could stably recapitulate relevant mechanical properties in cirrhotic liver tissue (81). The dECM of native liver consists of a wide range of glycosaminoglycans, proteins, collagens, and growth factors, providing a complex microenvironment to better support functionality and viability of liver cells and differentiation of liver progenitor compared with simple protein matrices used in common 2D or 3D cell culture models.

## 4 3D bioprinting of HPB and other digestive tissue and organs

The recent development of 3D bioprinting technology has provided various powerful 3D tissue models for HBP and other digestive organs, and it meets the demand for regenerative tissue and organ, which will improve patients’ life quality. This section reviews the recent studies on 3D bioprinted tumor models of HPB and other digestive organs. Ex vivo tumor models can offer help to the design of personalized treatment and to the understanding of mechanisms that underlie tumors. Notably, recreating the complexity of tumor microenvironment (TME) *in vitro* is important. The TME includes immune interactions, stroma, angiogenesis, cancer-associated fibroblasts (CAF) and ECM. The TME not only physically supports tumor growth but also promotes tumor development and metastasis (74, 82). Therefore, when we use 3D bioprinting to create HPB cancer models, we should importantly consider the factors in the TME.

### 4.1 Hepatocellular carcinoma

The liver plays a crucial role in digestion, metabolism and detoxification process, therefore severe liver disorders such as HCC and liver cirrhosis lead to a considerable threat to human health. HCC is the most prevalent primary liver cancer (3, 83). The majority of HCC tumors occur on the basis of cirrhosis or fibrosis, which are mainly caused by hepatitis B virus and hepatitis C virus infection, non-alcoholic fatty liver disease and alcohol-associated liver disease (83). Liver transplantation is a therapeutic solution for HCC patients at a certain disease stage, which is however limited by the shortage of donors and expenses. Fabrication of an *in vitro* liver model which can recapitulate the native liver microarchitecture is crucial for exploring hepatic metabolic functions, disease mechanisms, pathophysiology of hepatotoxicity and its accurate prediction, and personalized medicine. In liver tissue, hepatic lobules are the basic structures and functional units. It is still a challenge to recapitulate the small and complex structure and function of hepatic lobules *in vitro (* 84). The development of new technologies for liver bio-fabrication, especially 3D bioprinting, has attracted a great deal of attention owing to its promising ability to arrange the cells and biomaterials into the complex constructs similar to the native tissue (85).

HCC is an immunogenic cancer; it is capable of soliciting an immune response by virtue of its expression of tumor-associated antigens and neoantigens that can be recognized as ‘foreign’ entities (86). The tumor immune microenvironment comprises different immune cells. Both adaptive and innate cytotoxic cells can have tumor cell-killing function. CD4^+^ and CD8^+^ effector T cells play a key role in the anti-tumor process *via* different mechanisms. Antigen-presenting cells also play a key role in maintaining adaptive immune response in the tumor, such as intra-tumoral dendritic cells. Immunosuppressive cell populations such as regulatory T cells can also exist in the tumor immune microenvironment (87). HCC is immunogenic, but effective immune-mediated tumor control is prevented by its immunosuppressive tumor microenvironment (86). We found that tumor-infiltrating CD4^+^ and CD8^+^ T cells in HCC patients were functionally compromised. CD4^+^ and CD8^+^ T cells isolated from HCC tissue expressed certain co-inhibitory and co-stimulatory receptors, while dendritic cells, monocytes, and B cells in HCC tumors expressed ligands for these receptors (88, 89).

A 3D model *in vitro* with HCC cell line HepG2 cells (3DP-HepG2) was created using the extrusion-based 3D bioprinting technology and its biological activities were evaluated. The researchers found that 3DP-HepG2 showed notably increased expression of genes associated with tumor, such as CD133, CD24, ALB, AFP, IL-8, EpCAM and TGF-β genes, compared with 2D culture. The transcriptomic sequencing data indicated that 3DP-HepG2 model showed higher expression levels of genes related to liver functions than 2D-HepG2 model, including protein synthesis, lipid metabolism and glycogen metabolism, which suggests a possibility that the 3D printed microenvironment can support further differentiation of HepG2 cells (28). Moreover, the effects of anti-tumor drugs including cisplatin, sorafenib and regorafenib were compared in 2DP-HepG2 and 3DP-HepG2. They found the IC50 values obtained from the 3DP-HepG2 were closer to the effective blood concentrations of these drugs in human body, which may be associated with the increased expression of drug resistance and autophagy related genes in the 3DP-HepG2, including ACBC1, MDR-1, MRP1 and EGFR genes (28).

Furthermore, patient-derived 3D bioprinting hepatocellular carcinoma (3DP-HCC) models have been established. The researchers collected HCC specimens from six HCC patients after surgical resection, and they used extrusion-based 3D bioprinting technology to print 3D models with 10mm in length,10mm in with and 1.2mm in height. They proved that these models retained the features and tumorigenic potential of patient-derived HCC, including typical cell mass structure and expression of biomarkers such as AFP and Ki-67. They evaluated the efficacy of four empirical targeted drugs in these 3DP-HCC models, and found good agreement between sensitivities of the tested drugs and mutant targets revealed by whole exon sequencing (WES), which indicates that 3DP-HCC is a suitable personalized model for anti-HCC drug screening (29).

A new idea of combining 3D bioprinting with microfluidics was proposed, which was for pharmacodynamic test of an anti-CD147 monoclonal antibody, Metuzumab. This research provided results of migration and proliferation of human HCC SMMC-7721 cells and protein expression level on these cells after treating with the antibody, and found that Metuzumab could specifically bind to CD147, leading to secretion of MMP, and eventually result in a decrease in the invasive capacity of hepatoma cells. Especially, there was evidence that antibody-dependent cell-mediated cytotoxicity (ADCC) showed higher effectiveness in the microfluidic 3D bioprinting model than in the 3D bioprinting model without microfluidics (30).

Furthermore, liver sinusoid-like model has also been developed by coaxial 3D bioprinting (46). The model consisted of core pre-vascular structures and a shell compartment with hepatocytes. In this model, human endothelial cells and human fibroblasts in the core formed the pre-vascular network, supporting the HepG2 cells mechanically and biologically. The cellular interactions occurred in this triple co-culture model, which supplied a part of microenvironment to tumor development and metastasis. In addition, a physiologically relevant human vascularized liver model was bioprinted laden with human umbilical vein endothelial cells (HUVEC), human adipose mesenchymal stem cell-derived hepatocyte-like cells (HLC), and human hepatic stellate cells (HHSC) by using the extrusion-based bioprinting method (90). This HLC/HUVEC/HHSC-laden liver model resembled cords of hepatocytes with the functional sinusoidal lumen-like network, demonstrating enhanced urea synthesis, albumin production, and cytochrome P450 activity. These results would aid researchers to speed up the development of 3D HCC model with complicated microenvironment. Moreover, human-induced pluripotent stem cells (hiPSC) can be used as a promising cell source of bioink for the generation of functional hepatocytes (91).

### 4.2 Biliary cancer

The most common biliary malignancy is cholangiocarcinoma (CCA), intra-hepatic CCA is also the second most common primary liver malignancy, accounting for 10-15% of primary liver cancers (92). CCA has a typical feature of desmoplasia, with the presence of abundant fibrotic stroma infiltrating the tumor and a tumor immune microenvironment infiltrated with different immune cells (93). We found that proportions of cytotoxic T cells and natural killer cells were decreased, whereas regulatory T cells were increased in CCA tumors compared with tumor-free liver tissue from the same patients. While regulatory T cells accumulated in tumors, the majority of cytotoxic and helper T cells were sequestered at tumor margins, and natural killer cells were excluded from the tumors. The decreased numbers of cytotoxic immune cells and increased numbers of suppressor T cells that overexpress co-inhibitory receptors suggest that the tumor microenvironment in CCA is immunosuppressive (86, 94). The 3D CCA organoid model simulating tumor immune microenvironment could direct immunotherapy and predict prognosis (95).

3D bioprinting technology is suitable for being used to mimic CCA with complex desmoplasia, which cannot be obtained from 2D cultures or other 3D cultures. Direct mixing of stromal cells and tumor cells in a 3D microenvironment can lead to inhibition of contact between two types of cells during growth (96). Recently, Li et al. successfully constructed a 3D bioprinting immune microenvironment model of CCA by depositing cancer cells surrounded by stromal cells (**Figure 2B**). They compared the biological performance of tumor-associated endothelial cells (TEC), tumor-associated macrophages (TAM), and tumor-associated fibroblasts (TAF) in 3D immune microenvironment, and the results suggest that the stromal cells can promote tumor cell activity in 3D models. Gene expression levels of 3D bioprinted models in drug resistance, cancer stemness and proliferation were over 2-fold higher than those of 2D models. The enhanced cell malignancy in 3D bioprinted microenvironment also changed the expression of proteins associated with the epithelial-mesenchymal transition (EMT) process, including increased expression of MMP9, N-cadherin and vimentin and decreased expression of E-cadherin, which indicates that 3D bioprinting can promote the migration and invasion of tumors (65).

For intra-hepatic CCA model, markers including CK19, CK7, CK20, CEA, CDX2, CD15, CD133, CA19-9, MUC1, MUC2, S100P, TFF1, CD56, N-cadherin and EpCAM can be used to identify cancer type and cancer progression (97–104). For HCC model, markers including CK8, CK18, HepPar-1, Arg-1, AFP, HSP70, GPC3, CEA, CD24, CD133 and EpCAM can be used to identify cancer type and cancer progression (29, 30, 105–109). Because of the importance of immune cells, measuring proinflammatory cytokine levels, such as IL-1, IL-2, IL-12, IL-17, IL-18, IFN-γ and TNF-α (110, 111), is recommended for these 3D bioprinted tumor models and may help to gain more understanding of the immune microenvironment.

### 4.3 Pancreatic cancer

Pancreatic ductal adenocarcinoma (PDAC), a rapidly progressing disease, is the most frequent malignancy of pancreas with a high mortality. Both pancreatitis and pancreatic cancer share the same pathological pattern, in which exocrine pancreatic acinar cells transdifferentiate to ductal cells (112). This process triggered the application of 3D restructuration of pancreatic tissue. To define the mechanism underlying this process, Hakobyan et al. generated a 3D pancreatic spheroid model by using laser-assisted 3D bioprinting. The model consisted of both acinar and ductal cells and it could replicate the initial stages of PDAC (55). Moreover, 3D bioprinted pancreas is a promising treatment for patients who have insulin secretion deficiency and patients with pancreatic cancer who have secondary insulin deficiency after pancreatectomy. Recently, Salg et al. proposed the end-to-end concept from molecular level to macroscopic level to provide experimental proof for evaluating function of 3D bioprinting hybrid scaffold for secreting insulin (33). In this study, they printed pseudoislets from the rat INS-1 832/3 cell line as a viable and proliferative experimental model and found that co-culture of INS-I cells with endothelial cells enhanced insulin secretion (33).

### 4.4 Gastrointestinal cancers

Colorectal cancer (CRC) is the third most common malignancy with poor prognosis, accounting for about 10% of all newly diagnosed cancers (3). Therefore, it is an unmet need to develop more effective therapeutic strategies for CRC. However, the probability of a new drug passing from bench to bedside is below 0.1% (113). Sbirkov et al. established a 3D bioprinted CRC model by using Caco-2 cells as an economic and reproducible chemotherapeutic screening platform. They tested three commonly used chemotherapies including 5-fluorouracil, oxaliplatin, and irinotecan in this 3D model, and found increased drug resistance to 5-fluoruracil and irinotecan compared to conventional 2D culture. Of note, increased resistance to oxaliplatin was not observed, which might be related to the mode of action of this drug. The RNA-seq analysis provided a possible explanation for drug resistance that genes related to cell cycle progression were downregulated, genes related to hypoxia were upregulated, and TNF-α (via NF-κB) signature was enriched in 3D bioprinted model. This study also validated the proposed model with patient-derived CRC cells (**Figure 2D**) (14). Acoustic bioprinting was employed to print co-culture micro-tissue of colorectal cancer-cancer-associated fibroblasts (CRC-CAF) for modeling 3D cancer invasion and studying migration mechanisms (114). By observing the invasive dynamics and analyzing related protein expression of this CRC-CAF model, the invasive ability of this model was in accordance with the corresponding clinical data reflecting invasive ability of cancer cells, including data from pathological hematoxylin and eosin staining, immunohistochemistry and nuclear magnetic resonance imaging (MRI). Intestinal villus has also been bioprinted and showed expression of functional biomarkers including ZO-1 and villin, and such an intestinal villus model is promisingly applied to tumor models with vertical structures (45).

## 5 Limitations of 3D bioprinting and bioinks in clinical translation

Although 3D bioprinting is a state-of-the-art technology with a lot of potential, it still has some limitations in clinical translation. Firstly, 3D bioprinting remains limiting in producing tissue-like and biofunctional structures due to a shortage of optimized materials for bioinks. The suitable materials should provide structure support and mechanical protection in the process of printing (115). Additionally, the materials should also be biocompatible, bio-printable, nontoxic and degradable in the human body (22). However, early studies usually used viscous polymers which often lack cell compatibility and biofunction. Several studies have used alginate-collagen hydrogel (116), decellularized liver extracellular matrix (dLECM) hydrogel (117), silk protein (118), and nanocomposite materials (119) as bioinks, demonstrating better cytocompatibility, fidelity and support for tissue growth. Secondly, to meet the demand of clinical usage, the bioprinting process and products need to be evaluated under standard guidelines. Thirdly, it is important to remain enough live cells after the process of bioprinting for obtaining live tissue. Inkjet-based and laser-based bioprinting techniques have cell viability output over 85%, while extrusion-based bioprinting techniques have 40%-80% cell viability (120, 121). By reason of the complexity of cellular microenvironment, mimicking of cellular types, ECM and distributions is critical. Although strategies like extrusion-based bioprinting can be designed with different printing heads and multiple cell types, it is still a challenge to achieve precise position and distribution of the cells and biomaterials. The improvement of bioprinting strategies may increase the cell viability and enhance the precise control of constructing cellular microenvironment.

Moreover, standardizing the 3D bioprinted models to make decisions in personalized medicine-based clinical practice and drug evaluation that are most suitable and effective for individual patient’s specific type of cancer, as well as constructing models with patients’ own cells are challenging. Tumors from different patients have different biochemical components and structures, a combination of bioprinting techniques is required to accurately simulate individualized cancer models. For instance, in the process of recreating the complexity of cancer microenvironment *in vitro*, including stroma and tumor cell interaction, angiogenesis, and ECM remodeling, coaxial bioprinting can be used to develop microvasculature and extrusion-based bioprinting of dECM inks can be used to mimic the tumor-stroma interaction. When creating individualized cancer models for patients suffered from tumors with high density and high fibrosis, acoustic bioprinting can be a suitable option.

## 6 Future perspectives

As an innovative technology, 3D bioprinting has the potential to achieve a clinical application in HPB cancers and other diseases. Currently, a large number of commercial bioprinters have been developed for pharmaceutical companies and research institutes, and most of them are extrusion-based bioprinter for the reason of affordability and simplicity (122). It is urgent to establish standardized quality-control methods. We can have an ambitious idea that the living tissue can be directly printed and transplanted into the defective site in the operating room, which requires a sterile operating space with an incorporated bioprinter (123). Besides, there are numerous examples in which 3D bioprinted tumor models were used to tackle biological questions and design personalized therapies, but there are still a lot of possibilities and opportunities to explore. Advances of 3D bioprinting technologies in HPB cancer provide clinically relevant modeling capacity to create biomimetic, integrative, and human-based models. These models potentially recapitulate tissue-specific and species-matched features, including dimensionality, cell–cell interactions, and cell–matrix interactions of their biochemical and biomechanical counterparts. These models will be capable to serve as reliable *in vitro* models for screening of multiple candidate drugs for personalized treatment of patients with HPB cancer. Future investigation should be focused on the bioprinted models in clinical practice, which would require a different focus on technology directed towards the transplantation of bio-fabricated organs. Furthermore, bioprinting is moving beyond 3D to the emergence of 4D, and the term “time” as the fourth dimension is added. The human tissue has unique biofunction based on the dynamic changes of tissue induced by external stimuli. In the process of 4D bioprinting, materials can make a response to the stimulus after finishing the manufacturing (124). In other words, the 4D bioprinted constructs can change their structure and conformation with time induced by external stimuli under control of programs. The external stimuli include pH, water, temperature, pressure, electricity, magnetic field, drug and their combinations (18).

The merging of 3D bioprinting with other cutting-edge technologies may lead to a technological innovation enabling the development of increasingly intricate bio-fabrication that mimics complex native tissue. A microfluidic platform, enabling the generation of small liquid volume, can be utilized for generating multicellular spheroids and various cell assays, and has several advantages: 1) integrated components for removing waste and supplying nutrients (125), 2) integrated generators for concentration gradient drug delivery (126), 3) high-throughput drug screening with low cost (127), 4) automatic manufacturing processing instead of mass handling (128), 5) uniform spheroids. Multicellular spheroids produced by microfluidic platform are promising to serve as bioinks for high-cell-density 3D bioprinting. The developmental morphogenesis is based on coordinated cellular collective process mediated by cell-cell contact. Many diseases like fibrosis or tumors cannot be faithfully recapitulated with biomaterials in which single cells are dispersed throughout gels (74). Cell spheroids have been widely used as culture models *in vitro* benefiting from the high cell density and the potential to support cell sorting and differentiation, which can potThe control of size and uniformity of spheroids is important for maintaining optimal biological functions. However, the conventional methods for spheroids generation have limited control on uniformity and are not scaled for mass production in a high-throughput manner, which can be overcome by microfluidics. This may lead to the development of a hybrid bioprinting technology. Moreover, continuous dynamic perfusion of microfluidic technology can also be considered to supply nutrients and remove waste for uniform 3D bioprinted culture models, which can be conceived as a 3D microfluidic model. A 3D microfluidic model can be applied to pharmacodynamic tests and immunotherapeutic studies. Recent work demonstrated that a 3D microfluidic hepatoma model had been built (**Figure 2A**). The hepatoma cells in this model were found to have a higher proliferation efficiency than those in common 3D bioprinted models. This model was applied to test a new anti-CD147 monoclonal antibody and peripheral blood mononuclear cells were added through microfluidic channel as effector cells. The drug test results were in line with those obtained from animal models and clinical trials using similar anti-CD147 antibodies (30).

Different bioprinting methods allow the assembly of various types of cells suspended within different biomaterials, constructing a heterogeneous tissue-like model, as in the tumor microenvironment. A 3D bioprinted glioblastoma (GBM) model was fabricated to study the role of immune cells as a stromal component in tumor microenvironment by developing a co-culture model of GBM cells and GBM-associated macrophages within GelMA-based bioink (129). The model was a long-term culture model for real-time monitoring and able to mimic various juxtacrine, paracrine, and autocrine signaling pathways activated between tumor and stromal immune cells. In recent research, by embedding the 3D bioprinted tumoroids in an immune cell-laden collagen matrix, researchers could track the interaction between tumoroids and immune cells and study immunotherapy subsequently (130). In addition, a coaxial bioprinting model was developed for analysis of the interaction between macrophages and MDA-MB 231 breast cancer cells. In this research, breast cancer cells and macrophages were loaded into individual cartridges of the bioprinter and coextruded in a single step (131). In this printing process, the breast cancer cells were embedded in alginate-based bioink and extruded through the outer channel, and the macrophages were suspended in the CaCl_2_ solution and delivered *via* the inner channel to cross-link the outer alginate flow. It was found that the macrophages initially existing in the inner channel gradually migrated out and had interaction with the surrounding breast cancer cells. Currently, 3D co-cultures of tumor organoids and immune cells including lymphocytes have been established (95), using 3D bioprinting technology to simulate a more complete and complex tumor immune microenvironment is anticipated.

## Author contributions

Conception and design: XZ and GZ. Manuscript writing: XZ and GZ. Revision of manuscript: All the authors. All authors contributed to the article and approved the submitted version.

## References

[1] Yu J Refsum E Helsingen LM Folseraas T Ploner A Wieszczy P. Risk of hepato-Pancreato-Biliary cancer is increased by primary sclerosing cholangitis in patients with inflammatory bowel disease: a population-based cohort study. United Eur Gastroenterol J (2022) 10(2):212–24. doi: 10.1002/ueg2.12204 PMC8911542 35107865

[2] Bray F Ferlay J Soerjomataram I Siegel RL Torre LA Jemal A. Global cancer statistics 2018: globocan estimates of incidence and mortality worldwide for 36 cancers in 185 countries. CA: Cancer J Clin (2018) 68(6):394–424. doi: 10.3322/caac.21492 30207593

[3] Sung H Ferlay J Siegel RL Laversanne M Soerjomataram I Jemal A. Global cancer statistics 2020: globocan estimates of incidence and mortality worldwide for 36 cancers in 185 countries. CA: Cancer J Clin (2021) 71(3):209–49. doi: 10.3322/caac.21660 33538338

[4] Akram P Sadaf GS Kevin SI Catherine B Saeid S Gholamreza R. The global, regional, and national burden of pancreatic cancer and its attributable risk factors in 195 countries and territories, 1990-2017: a systematic analysis for the global burden of disease study 2017. Lancet Gastroenterol Hepatol (2019) 4(12):934–47. doi: 10.1016/s2468-1253(19)30347-4 PMC7026711 31648972

[5] Yuan B Zhao X Wang X Liu E Liu C Zong Y. Patient-derived organoids for personalized gallbladder cancer modelling and drug screening. Clin Trans Med (2022) 12(1):e678. doi: 10.1002/ctm2.678 PMC8786696 35075805

[6] Siegel RL Miller KD Fuchs HE Jemal A. Cancer statistics, 2022. CA: Cancer J Clin (2022) 72(1):7–33. doi: 10.3322/caac.21708 35020204

[7] Hickman L Contreras C. Gallbladder cancer: diagnosis, surgical management, and adjuvant therapies. Surg Clinics North America (2019) 99(2):337–55. doi: 10.1016/j.suc.2018.12.008 30846038

[8] Zhang B Zhou J Xie W Tao K Lu S Yuan X. Expert consensus on organizing the multidisciplinary team (Mdt) diagnosis and treatment of hepato-Pancreato-Biliary diseases in China. Sci China Life Sci (2022) 65(5):1036–9. doi: 10.1007/s11427-021-2079-7 35314917

[9] Sangro B Sarobe P Hervás-Stubbs S Melero I. Advances in immunotherapy for hepatocellular carcinoma. Nat Rev Gastroenterol Hepatol (2021) 18(8):525–43. doi: 10.1038/s41575-021-00438-0 PMC8042636 33850328

[10] El-Khoueiry AB Sangro B Yau T Crocenzi TS Kudo M Hsu C. Nivolumab in patients with advanced hepatocellular carcinoma (Checkmate 040): an open-label, non-comparative, phase 1/2 dose escalation and expansion trial. Lancet (London England) (2017) 389(10088):2492–502. doi: 10.1016/s0140-6736(17)31046-2 PMC7539326 28434648

[11] Zhu AX Finn RS Edeline J Cattan S Ogasawara S Palmer D. Pembrolizumab in patients with advanced hepatocellular carcinoma previously treated with sorafenib (Keynote-224): a non-randomised, open-label phase 2 trial. Lancet Oncol (2018) 19(7):940–52. doi: 10.1016/s1470-2045(18)30351-6 29875066

[12] Tsilimigras DI Brodt P Clavien PA Muschel RJ D’Angelica MI Endo I. Liver metastases. Nat Rev Dis Primers (2021) 7(1):27. doi: 10.1038/s41572-021-00261-6 33859205

[13] Mazzaferro V Citterio D Bhoori S Bongini M Miceli R De Carlis L. Liver transplantation in hepatocellular carcinoma after tumour downstaging (Xxl): a randomised, controlled, phase 2b/3 trial. Lancet Oncol (2020) 21(7):947–56. doi: 10.1016/s1470-2045(20)30224-2 32615109

[14] Sbirkov Y Molander D Milet C Bodurov I Atanasov B Penkov R. A colorectal cancer 3d bioprinting workflow as a platform for disease modeling and chemotherapeutic screening. Front Bioeng Biotechnol (2021) 9:755563. doi: 10.3389/fbioe.2021.755563 34869264 PMC8638705

[15] Drost J Clevers H. Organoids in cancer research. Nat Rev Cancer (2018) 18(7):407–18. doi: 10.1038/s41568-018-0007-6 29692415

[16] Tuveson D Clevers H. Cancer modeling meets human organoid technology. Sci (New York NY) (2019) 364(6444):952–5. doi: 10.1126/science.aaw6985 31171691

[17] Langer EM Allen-Petersen BL King SM Kendsersky ND Turnidge MA Kuziel GM. Modeling tumor phenotypes *In vitro* with three-dimensional bioprinting. Cell Rep (2019) 26(3):608–23.e6. doi: 10.1016/j.celrep.2018.12.090 30650355 PMC6366459

[18] Heinrich MA Liu W Jimenez A Yang J Akpek A Liu X. 3d bioprinting: from benches to translational applications. Small (Weinheim an der Bergstrasse Germany) (2019) 15(23):e1805510. doi: 10.1002/smll.201805510 31033203 PMC6752725

[19] Zhang YS Yue K Aleman J Moghaddam KM Bakht SM Yang J. 3d bioprinting for tissue and organ fabrication. Ann Biomed Eng (2017) 45(1):148–63. doi: 10.1007/s10439-016-1612-8 PMC5085899 27126775

[20] Murphy SV Atala A. 3d bioprinting of tissues and organs. Nat Biotechnol (2014) 32(8):773–85. doi: 10.1038/nbt.2958 25093879

[21] Matai I Kaur G Seyedsalehi A McClinton A Laurencin CT. Progress in 3d bioprinting technology for Tissue/Organ regenerative engineering. Biomaterials (2020) 226:119536. doi: 10.1016/j.biomaterials.2019.119536 31648135

[22] Li J Chen M Fan X Zhou H. Recent advances in bioprinting techniques: approaches, applications and future prospects. J Trans Med (2016) 14:271. doi: 10.1186/s12967-016-1028-0 PMC5028995 27645770

[23] Mahadik B Margolis R McLoughlin S Melchiorri A Lee SJ Yoo J. An open-source bioink database for microextrusion 3d printing. Biofabrication (2022) 15(1). doi: 10.1088/1758-5090/ac933a PMC9652762 36126638

[24] Freeman S Calabro S Williams R Jin S Ye K. Bioink formulation and machine learning-empowered bioprinting optimization. Front Bioeng Biotechnol (2022) 10:913579. doi: 10.3389/fbioe.2022.913579 35782492 PMC9240914

[25] An J Chua CK Mironov V. Application of machine learning in 3d bioprinting: focus on development of big data and digital twin. Int J bioprinting (2021) 7(1):342. doi: 10.18063/ijb.v7i1.342 PMC7875058 33585718

[26] Ruberu K Senadeera M Rana S Gupta S Chung JHY Yue Z. Coupling machine learning with 3d bioprinting to fast track optimisation of extrusion printing. Appl Mater Today (2021) 22:100914. doi: 10.1016/j.apmt.2020.100914

[27] Banerjee D Singh YP Datta P Ozbolat V O’Donnell A Yeo M. Strategies for 3d bioprinting of spheroids: a comprehensive review. Biomaterials (2022) 291:121881. doi: 10.1016/j.biomaterials.2022.121881 36335718

[28] Sun L Yang H Wang Y Zhang X Jin B Xie F. Application of a 3d bioprinted hepatocellular carcinoma cell model in antitumor drug research. Front Oncol (2020) 10:878. doi: 10.3389/fonc.2020.00878 32582546 PMC7283506

[29] Xie F Sun L Pang Y Xu G Jin B Xu H. Three-dimensional bio-printing of primary human hepatocellular carcinoma for personalized medicine. Biomaterials (2021) 265:120416. doi: 10.1016/j.biomaterials.2020.120416 33007612

[30] Li Y Zhang T Pang Y Li L Chen ZN Sun W. 3d bioprinting of hepatoma cells and application with microfluidics for pharmacodynamic test of metuzumab. Biofabrication (2019) 11(3):034102. doi: 10.1088/1758-5090/ab256c 31141796

[31] Gao Q He Y Fu JZ Liu A Ma L. Coaxial nozzle-assisted 3d bioprinting with built-in microchannels for nutrients delivery. Biomaterials (2015) 61:203–15. doi: 10.1016/j.biomaterials.2015.05.031 26004235

[32] Zhang Y Yu Y Akkouch A Dababneh A Dolati F Ozbolat IT. *In vitro* study of directly bioprinted perfusable vasculature conduits. Biomater Sci (2015) 3(1):134–43. doi: 10.1039/c4bm00234b PMC4283831 25574378

[33] Salg GA Poisel E Neulinger-Munoz M Gerhardus J Cebulla D Bludszuweit-Philipp C. Toward 3d-bioprinting of an endocrine pancreas: a building-block concept for bioartificial insulin-secreting tissue. J Tissue Eng (2022) 13:20417314221091033. doi: 10.1177/20417314221091033 35462988 PMC9024162

[34] Chirivì M Bearzi C Rosa P Miglietta S Petronella F De Falco E. Biomimetic keratin-coated gold nanoparticles for photo-thermal therapy in a 3d bioprinted glioblastoma tumor model. Int J Mol Sci (2022) 23(17):9528. doi: 10.3390/ijms23179528 36076927 PMC9455633

[35] Sorkio A Koch L Koivusalo L Deiwick A Miettinen S Chichkov B. Human stem cell based corneal tissue mimicking structures using laser-assisted 3d bioprinting and functional bioinks. Biomaterials (2018) 171:57–71. doi: 10.1016/j.biomaterials.2018.04.034 29684677

[36] Kawecki F Clafshenkel WP Auger FA Bourget JM Fradette J Devillard R. Self-assembled human osseous cell sheets as living biopapers for the laser-assisted bioprinting of human endothelial cells. Biofabrication (2018) 10(3):035006. doi: 10.1088/1758-5090/aabd5b 29638221

[37] Solis LH Ayala Y Portillo S Varela-Ramirez A Aguilera R Boland T. Thermal inkjet bioprinting triggers the activation of the vegf pathway in human microvascular endothelial cells in vitro. Biofabrication (2019) 11(4):045005. doi: 10.1088/1758-5090/ab25f9 31151129 PMC7244212

[38] Takagi D Lin W Matsumoto T Yaginuma H Hemmi N Hatada S. High-precision three-dimensional inkjet technology for live cell bioprinting. Int J bioprinting (2019) 5(2):208. doi: 10.18063/ijb.v5i2.208 PMC7294685 32596539

[39] Koch L Deiwick A Schlie S Michael S Gruene M Coger V. Skin tissue generation by laser cell printing. Biotechnol Bioeng (2012) 109(7):1855–63. doi: 10.1002/bit.24455 22328297

[40] Xu C Chai W Huang Y Markwald RR. Scaffold-free inkjet printing of three-dimensional zigzag cellular tubes. Biotechnol Bioeng (2012) 109(12):3152–60. doi: 10.1002/bit.24591 22767299

[41] Souza AG Silva IBB Campos-Fernandez E Barcelos LS Souza JB Marangoni K. Comparative assay of 2d and 3d cell culture models: proliferation, gene expression and anticancer drug response. Curr Pharm design (2018) 24(15):1689–94. doi: 10.2174/1381612824666180404152304 29623827

[42] Daquinag AC Souza GR Kolonin MG. Adipose tissue engineering in three-dimensional levitation tissue culture system based on magnetic nanoparticles. Tissue Eng Part C Methods (2013) 19(5):336–44. doi: 10.1089/ten.TEC.2012.0198 PMC3603558 23017116

[43] Tseng H Gage JA Raphael RM Moore RH Killian TC Grande-Allen KJ. Assembly of a three-dimensional multitype bronchiole coculture model using magnetic levitation. Tissue Eng Part C Methods (2013) 19(9):665–75. doi: 10.1089/ten.TEC.2012.0157 23301612

[44] Tseng H Balaoing LR Grigoryan B Raphael RM Killian TC Souza GR. A three-dimensional Co-culture model of the aortic valve using magnetic levitation. Acta biomaterialia (2014) 10(1):173–82. doi: 10.1016/j.actbio.2013.09.003 PMC10593146 24036238

[45] Lian L Zhou C Tang G Xie M Wang Z Luo Z. Uniaxial and coaxial vertical embedded extrusion bioprinting. Advanced healthcare mater (2022) 11(9):e2102411. doi: 10.1002/adhm.202102411 34860472

[46] Taymour R Chicaiza-Cabezas NA Gelinsky M Lode A. Core-shell bioprinting of vascularizedin vitroliver sinusoid models. Biofabrication (2022) 14(4). doi: 10.1088/1758-5090/ac9019 36070706

[47] Jentsch S Nasehi R Kuckelkorn C Gundert B Aveic S Fischer H. Multiscale 3d bioprinting by nozzle-free acoustic droplet ejection. Small Methods (2021) 5(6):e2000971. doi: 10.1002/smtd.202000971 34927902

[48] Chen K Jiang E Wei X Xia Y Wu Z Gong Z. The acoustic droplet printing of functional tumor microenvironments. Lab chip (2021) 21(8):1604–12. doi: 10.1039/d1lc00003a 33683268

[49] Kačarević Ž Rider PM Alkildani S Retnasingh S Smeets R Jung O. An Introduction to 3d Bioprinting: Possibilities, Challenges and Future Aspects. Materials (Basel, Switzerland) (2018) 11(11):2199. doi: 10.3390/ma11112199 30404222 PMC6266989

[50] Xu K Han Y Huang Y Wei P Yin J Jiang J. The application of 3d bioprinting in urological diseases. Mater Today Bio (2022) 16:100388. doi: 10.1016/j.mtbio.2022.100388 PMC9364106 35967737

[51] Xiaorui L Fuyin Z Xudong W Xuezheng G Shudong Z Hui L. 1biomaterial Inks for Extrusion-Based 3d Bioprinting: Property, Classification, Modification, and Selection. International journal of bioprinting (2023) 9(2):649. doi: 10.18063/ijb.v9i2.649 37065674 PMC10090818

[52] Hong G Kim J Oh H Yun S Kim CM Jeong YM. Production of multiple cell-laden microtissue spheroids with a biomimetic hepatic-Lobule-Like structure. Advanced mater (Deerfield Beach Fla) (2021) 33(36):e2102624. doi: 10.1002/adma.202102624 PMC11469225 34286875

[53] You F Eames BF Chen X. Application of extrusion-based hydrogel bioprinting for cartilage tissue engineering. Int J Mol Sci (2017) 18(7):1597. doi: 10.3390/ijms18071597 28737701 PMC5536084

[54] Barron JA Wu P Ladouceur HD Ringeisen BR. Biological laser printing: a novel technique for creating heterogeneous 3-dimensional cell patterns. Biomed microdevices (2004) 6(2):139–47. doi: 10.1023/b:bmmd.0000031751.67267.9f 15320636

[55] Hakobyan D Médina C Dusserre N Stachowicz ML Handschin C Fricain JC. Laser-assisted 3d bioprinting of exocrine pancreas spheroid models for cancer initiation study. Biofabrication (2020) 12(3):035001. doi: 10.1088/1758-5090/ab7cb8 32131058

[56] Yu J Park SA Kim WD Ha T Xin YZ Lee J. Current advances in 3d bioprinting technology and its applications for tissue engineering. Polymers (2020) 12(12). doi: 10.3390/polym12122958 PMC7764360 33322291

[57] Guillotin B Souquet A Catros S Duocastella M Pippenger B Bellance S. Laser assisted bioprinting of engineered tissue with high cell density and microscale organization. Biomaterials (2010) 31(28):7250–6. doi: 10.1016/j.biomaterials.2010.05.055 20580082

[58] Gudapati H Dey M Ozbolat I. A comprehensive review on droplet-based bioprinting: past, present and future. Biomaterials (2016) 102:20–42. doi: 10.1016/j.biomaterials.2016.06.012 27318933

[59] Derby B. Bioprinting: inkjet printing proteins and hybrid cell-containing materials and structures. J Mater Chem (2008) 18:5717–21. doi: 10.1039/b807560c

[60] Gao G Yonezawa T Hubbell K Dai G Cui X. Inkjet-bioprinted acrylated peptides and peg hydrogel with human mesenchymal stem cells promote robust bone and cartilage formation with minimal printhead clogging. Biotechnol J (2015) 10(10):1568–77. doi: 10.1002/biot.201400635 25641582

[61] Cui X Boland T. Human microvasculature fabrication using thermal inkjet printing technology. Biomaterials (2009) 30(31):6221–7. doi: 10.1016/j.biomaterials.2009.07.056 19695697

[62] Perçin G Khuri-Yakub BT. Piezoelectric droplet ejector for ink-jet printing of fluids and solid particles. Rev Sci Instruments (2003) 74:1120–7. doi: 10.1063/1.1532839

[63] Liu J Shahriar M Xu H Xu C. Cell-laden bioink circulation-assisted inkjet-based bioprinting to mitigate cell sedimentation and aggregation. Biofabrication (2022) 14(4). doi: 10.1088/1758-5090/ac8fb7 36067747

[64] Haisler WL Timm DM Gage JA Tseng H Killian TC Souza GR. Three-dimensional cell culturing by magnetic levitation. Nat Protoc (2013) 8(10):1940–9. doi: 10.1038/nprot.2013.125 24030442

[65] Li C Jin B Sun H Wang Y Zhao H Sang X. Exploring the function of stromal cells in cholangiocarcinoma by three-dimensional bioprinting immune microenvironment model. Front Immunol (2022) 13:941289. doi: 10.3389/fimmu.2022.941289 35983036 PMC9378822

[66] Fernandez-Vega V Hou S Plenker D Tiriac H Baillargeon P Shumate J. Lead identification using 3d models of pancreatic cancer. SLAS discovery: advancing Life Sci R D (2022) 27(3):159–66. doi: 10.1016/j.slasd.2022.03.002 PMC10258910 35306207

[67] Jia W Gungor-Ozkerim PS Zhang YS Yue K Zhu K Liu W. Direct 3d bioprinting of perfusable vascular constructs using a blend bioink. Biomaterials (2016) 106:58–68. doi: 10.1016/j.biomaterials.2016.07.038 27552316 PMC5300870

[68] Böttcher B Pflieger A Schumacher J Jungnickel B Feller KH. 3d bioprinting of prevascularized full-thickness gelatin-alginate structures with embedded Co-cultures. Bioeng (Basel Switzerland) (2022) 9(6). doi: 10.3390/bioengineering9060242 PMC9219913 35735485

[69] Mao M Liang H He J Kasimu A Zhang Y Wang L. Coaxial electrohydrodynamic bioprinting of pre-vascularized cell-laden constructs for tissue engineering. Int J bioprinting (2021) 7(3):362. doi: 10.18063/ijb.v7i3.362 PMC8287508 34286149

[70] Wu Z Cai H Ao Z Xu J Heaps S Guo F. Microfluidic printing of tunable hollow microfibers for vascular tissue engineering. Advanced mater Technol (2021) 6(8):2000683. doi: 10.1002/admt.202000683 PMC8386518 34458563

[71] Ozbolat IT Hospodiuk M. Current advances and future perspectives in extrusion-based bioprinting. Biomaterials (2016) 76:321–43. doi: 10.1016/j.biomaterials.2015.10.076 26561931

[72] Ahlfeld T Guduric V Duin S Akkineni AR Schütz K Kilian D. Methylcellulose – a versatile printing material that enables biofabrication of tissue equivalents with high shape fidelity. Biomater Sci (2020) 8(8):2102–10. doi: 10.1039/D0BM00027B 32236265

[73] Liu W Heinrich MA Zhou Y Akpek A Hu N Liu X. Extrusion bioprinting of shear-thinning gelatin methacryloyl bioinks. Advanced healthcare mater (2017) 6(12). doi: 10.1002/adhm.201601451 PMC5545786 28464555

[74] Daly AC Prendergast ME Hughes AJ Burdick JA. Bioprinting for the biologist. Cell (2021) 184(1):18–32. doi: 10.1016/j.cell.2020.12.002 33417859 PMC10335003

[75] Faulkner-Jones A Fyfe C Cornelissen DJ Gardner J King J Courtney A. Bioprinting of human pluripotent stem cells and their directed differentiation into hepatocyte-like cells for the generation of mini-livers in 3d. Biofabrication (2015) 7(4):44102. doi: 10.1088/1758-5090/7/4/044102 26486521

[76] Blandino A Macías M Cantero D. Formation of calcium alginate gel capsules: influence of sodium alginate and Cacl2 concentration on gelation kinetics. J biosci Bioeng (1999) 88(6):686–9. doi: 10.1016/s1389-1723(00)87103-0 16232687

[77] Nichol JW Koshy ST Bae H Hwang CM Yamanlar S Khademhosseini A. Cell-laden microengineered gelatin methacrylate hydrogels. Biomaterials (2010) 31(21):5536–44. doi: 10.1016/j.biomaterials.2010.03.064 PMC2878615 20417964

[78] Hutson CB Nichol JW Aubin H Bae H Yamanlar S Al-Haque S. Synthesis and characterization of tunable Poly(Ethylene glycol): gelatin methacrylate composite hydrogels. Tissue Eng Part A (2011) 17(13-14):1713–23. doi: 10.1089/ten.TEA.2010.0666 PMC3118706 21306293

[79] Park JY Choi JC Shim JH Lee JS Park H Kim SW. A comparative study on collagen type I and hyaluronic acid dependent cell behavior for osteochondral tissue bioprinting. Biofabrication (2014) 6(3):35004. doi: 10.1088/1758-5082/6/3/035004 24758832

[80] Ott HC Matthiesen TS Goh SK Black LD Kren SM Netoff TI. Perfusion-decellularized matrix: using nature’s platform to engineer a bioartificial heart. Nat Med (2008) 14(2):213–21. doi: 10.1038/nm1684 18193059

[81] Ma X Yu C Wang P Xu W Wan X Lai CSE. Rapid 3d bioprinting of decellularized extracellular matrix with regionally varied mechanical properties and biomimetic microarchitecture. Biomaterials (2018) 185:310–21. doi: 10.1016/j.biomaterials.2018.09.026 PMC6186504 30265900

[82] Spill F Reynolds DS Kamm RD Zaman MH. Impact of the physical microenvironment on tumor progression and metastasis. Curr Opin Biotechnol (2016) 40:41–8. doi: 10.1016/j.copbio.2016.02.007 PMC4975620 26938687

[83] Calderaro J Seraphin TP Luedde T Simon TG. Artificial intelligence for the prevention and clinical management of hepatocellular carcinoma. J Hepatol (2022) 76(6):1348–61. doi: 10.1016/j.jhep.2022.01.014 PMC9126418 35589255

[84] Zhang J Yang H Yang H. Highlights of constructing liver-relevant in vitro models with 3d bioprinting. Hepatob Surg Nutr (2022) 11(6):896–8. doi: 10.21037/hbsn-22-486 PMC9745620 36523922

[85] Groll J Boland T Blunk T Burdick JA Cho DW Dalton PD. Biofabrication: reappraising the definition of an evolving field. Biofabrication (2016) 8(1):13001. doi: 10.1088/1758-5090/8/1/013001 26744832

[86] Zhou G Boor PPC Bruno MJ Sprengers D Kwekkeboom J. Immune suppressive checkpoint interactions in the tumour microenvironment of primary liver cancers. Br J Cancer (2022) 126(1):10–23. doi: 10.1038/s41416-021-01453-3 34400801 PMC8727557

[87] Zhou G Noordam L Sprengers D Doukas M Boor PPC van Beek AA. Blockade of Lag3 enhances responses of tumor-infiltrating T cells in mismatch repair-proficient liver metastases of colorectal cancer. Oncoimmunology (2018) 7(7):e1448332. doi: 10.1080/2162402x.2018.1448332 29900067 PMC5993483

[88] Zhou G Sprengers D Boor PPC Doukas M Schutz H Mancham S. Antibodies against immune checkpoint molecules restore functions of tumor-infiltrating T cells in hepatocellular carcinomas. Gastroenterology (2017) 153(4):1107–19.e10. doi: 10.1053/j.gastro.2017.06.017 28648905

[89] Pedroza-Gonzalez A Zhou G Singh SP Boor PP Pan Q Grunhagen D. Gitr engagement in combination with ctla-4 blockade completely abrogates immunosuppression mediated by human liver tumor-derived regulatory T cells ex vivo. Oncoimmunology (2015) 4(12):e1051297. doi: 10.1080/2162402x.2015.1051297 26587321 PMC4635937

[90] Janani G Priya S Dey S Mandal BB. Mimicking native liver lobule microarchitecture in vitro with parenchymal and non-parenchymal cells using 3d bioprinting for drug toxicity and drug screening applications. ACS Appl mater interfaces (2022) 14(8):10167–86. doi: 10.1021/acsami.2c00312 35171571

[91] He J Wang J Pang Y Yu H Qin X Su K. Bioprinting of a hepatic tissue model using human-induced pluripotent stem cell-derived hepatocytes for drug-induced hepatotoxicity evaluation. Int J bioprinting (2022) 8(3):581. doi: 10.18063/ijb.v8i3.581 PMC9468961 36105133

[92] Alsaleh M Leftley Z Barbera TA Sithithaworn P Khuntikeo N Loilome W. Cholangiocarcinoma: a guide for the nonspecialist. Int J Gen Med (2019) 12:13–23. doi: 10.2147/ijgm.S186854 30588065 PMC6304240

[93] Fabris L Perugorria MJ Mertens J Björkström NK Cramer T Lleo A. The tumour microenvironment and immune milieu of cholangiocarcinoma. Liver Int (2019) 39 Suppl 1:63–78. doi: 10.1111/liv.14098 30907492 PMC10878127

[94] Zhou G Sprengers D Mancham S Erkens R Boor PPC van Beek AA. Reduction of immunosuppressive tumor microenvironment in cholangiocarcinoma by ex vivo targeting immune checkpoint molecules. J Hepatol (2019) 71(4):753–62. doi: 10.1016/j.jhep.2019.05.026 31195061

[95] Zhou G Lieshout R van Tienderen GS de Ruiter V van Royen ME Boor PPC. Modelling immune cytotoxicity for cholangiocarcinoma with tumour-derived organoids and effector T cells. Br J Cancer (2022) 127(4):649–60. doi: 10.1038/s41416-022-01839-x PMC9381772 35597867

[96] Lazzari G Nicolas V Matsusaki M Akashi M Couvreur P Mura S. Multicellular spheroid based on a triple Co-culture: a novel 3d model to mimic pancreatic tumor complexity. Acta biomaterialia (2018) 78:296–307. doi: 10.1016/j.actbio.2018.08.008 30099198

[97] Mao S He J Zhao Y Liu T Xie F Yang H. Bioprinting of patient-derived in vitro intrahepatic cholangiocarcinoma tumor model: establishment, evaluation and anti-cancer drug testing. Biofabrication (2020) 12(4):045014. doi: 10.1088/1758-5090/aba0c3 32599574

[98] Lee JI Lee JW Kim JM Kim JK Chung HJ Kim YS. Prognosis of hepatocellular carcinoma expressing cytokeratin 19: comparison with other liver cancers. World J Gastroenterol (2012) 18(34):4751–7. doi: 10.3748/wjg.v18.i34.4751 PMC3442214 23002345

[99] Li JY Wang LL Fan J Liu DX Han JB Zhang YF. New and effective method to develop primary hepatocytes from liver cancer patients. Exp Biol Med (Maywood NJ) (2022) 247(11):972–81. doi: 10.1177/15353702221085534 PMC9189565 35470702

[100] Zen Y Sasaki M Fujii T Chen TC Chen MF Yeh TS. Different expression patterns of mucin core proteins and cytokeratins during intrahepatic cholangiocarcinogenesis from biliary intraepithelial neoplasia and intraductal papillary neoplasm of the bile duct–an immunohistochemical study of 110 cases of hepatolithiasis. J Hepatol (2006) 44(2):350–8. doi: 10.1016/j.jhep.2005.09.025 16360234

[101] Walter D Herrmann E Winkelmann R Albert JG Liese J Schnitzbauer A. Role of Cd15 expression in dysplastic and neoplastic tissue of the bile duct - a potential novel tool for differential diagnosis of indeterminate biliary stricture. Histopathology (2016) 69(6):962–70. doi: 10.1111/his.13041 27442966

[102] Balitzer D Joseph NM Ferrell L Shafizadeh N Jain D Zhang X. Immunohistochemical and molecular features of cholangiolocellular carcinoma are similar to well-differentiated intrahepatic cholangiocarcinoma. Modern Pathol (2019) 32(10):1486–94. doi: 10.1038/s41379-019-0290-0 31186529

[103] Akita M Sawada R Komatsu M Suleman N Itoh T Ajiki T. An immunostaining panel of c-reactive protein, n-cadherin, and S100 calcium binding protein p is useful for intrahepatic cholangiocarcinoma subtyping. Hum Pathol (2021) 109:45–52. doi: 10.1016/j.humpath.2020.12.005 33321161

[104] Yeh TS Tseng JH Chen TC Liu NJ Chiu CT Jan YY. Characterization of intrahepatic cholangiocarcinoma of the intraductal growth-type and its precursor lesions. Hepatol (Baltimore Md) (2005) 42(3):657–64. doi: 10.1002/hep.20837 16116640

[105] Yan Q Lin HM Zhu K Cao Y Xu XL Zhou ZY. Immune checkpoint Fgl1 expression of circulating tumor cells is associated with poor survival in curatively resected hepatocellular carcinoma. Front Oncol (2022) 12:810269. doi: 10.3389/fonc.2022.810269 35273912 PMC8901582

[106] Lu SX Huang YH Liu LL Zhang CZ Yang X Yang YZ. A-fetoprotein mrna in situ hybridisation is a highly specific marker of hepatocellular carcinoma: a multi-centre study. Br J Cancer (2021) 124(12):1988–96. doi: 10.1038/s41416-021-01363-4 PMC8184895 33824478

[107] He Y Luo J Zhang G Jin Y Wang N Lu J. Single-cell profiling of human Cd127(+) innate lymphoid cells reveals diverse immune phenotypes in hepatocellular carcinoma. Hepatol (Baltimore Md) (2022) 76(4):1013–29. doi: 10.1002/hep.32444 PMC9790738 35243668

[108] Morrison C Marsh W Jr. Frankel WL. A comparison of Cd10 to pcea, moc-31, and hepatocyte for the distinction of malignant tumors in the liver. Modern Pathol (2002) 15(12):1279–87. doi: 10.1097/01.Mp.0000037312.69565.24 12481008

[109] Yang XR Xu Y Yu B Zhou J Li JC Qiu SJ. Cd24 is a novel predictor for poor prognosis of hepatocellular carcinoma after surgery. Clin Cancer Res (2009) 15(17):5518–27. doi: 10.1158/1078-0432.Ccr-09-0151 19706825

[110] Ma HY Yamamoto G Xu J Liu X Karin D Kim JY. Il-17 signaling in steatotic hepatocytes and macrophages promotes hepatocellular carcinoma in alcohol-related liver disease. J Hepatol (2020) 72(5):946–59. doi: 10.1016/j.jhep.2019.12.016 PMC7167339 31899206

[111] Cui J Chen Y Wang HY Wang RF. Mechanisms and pathways of innate immune activation and regulation in health and cancer. Hum Vaccines immunotherapeutics (2014) 10(11):3270–85. doi: 10.4161/21645515.2014.979640 PMC4514086 25625930

[112] Zhu L Shi G Schmidt CM Hruban RH Konieczny SF. Acinar cells contribute to the molecular heterogeneity of pancreatic intraepithelial neoplasia. Am J Pathol (2007) 171(1):263–73. doi: 10.2353/ajpath.2007.061176 PMC1941579 17591971

[113] Seyhan AA. Lost in translation: the valley of death across preclinical and clinical divide – identification of problems and overcoming obstacles. Trans Med Commun (2019) 4:1–19. doi: 10.1186/s41231-019-0050-7

[114] Chen H Du L Li J Wu Z Gong Z Xia Y. Modeling cancer metastasis using acoustically bio-printed patient-derived 3d tumor microtissues. J mater Chem B (2022) 10(11):1843–52. doi: 10.1039/d1tb02789a 35224593

[115] Hull SM Brunel LG Heilshorn SC. 3d bioprinting of cell-laden hydrogels for improved biological functionality. Advanced mater (Deerfield Beach Fla) (2022) 34(2):e2103691. doi: 10.1002/adma.202103691 PMC8988886 34672027

[116] Kim SD Jin S Kim S Son D Shin M. Tyramine-functionalized alginate-collagen hybrid hydrogel inks for 3d-bioprinting. Polymers (2022) 14(15):3173. doi: 10.3390/polym14153173 35956690 PMC9371113

[117] Khati V Turkki JA Ramachandraiah H Pati F Gaudenzi G Russom A. Indirect 3d bioprinting of a robust trilobular hepatic construct with decellularized liver matrix hydrogel. Bioeng (Basel Switzerland) (2022) 9(11):603. doi: 10.3390/bioengineering9110603 PMC9687301 36354514

[118] Chakraborty J Mu X Pramanick A Kaplan DL Ghosh S. Recent advances in bioprinting using silk protein-based bioinks. Biomaterials (2022) 287:121672. doi: 10.1016/j.biomaterials.2022.121672 35835001

[119] Cai Y Chang SY Gan SW Ma S Lu WF Yen CC. Nanocomposite bioinks for 3d bioprinting. Acta biomaterialia (2022) 151:45–69. doi: 10.1016/j.actbio.2022.08.014 35970479

[120] Xu T Jin J Gregory C Hickman JJ Boland T. Inkjet printing of viable mammalian cells. Biomaterials (2005) 26(1):93–9. doi: 10.1016/j.biomaterials.2004.04.011 15193884

[121] Catros S Guillotin B Bačáková M Fricain J-C Guillemot F. Effect of laser energy, substrate film thickness and bioink viscosity on viability of endothelial cells printed by laser-assisted bioprinting. Appl Surface Sci (2011) 257:5142–7. doi: 10.1016/j.apsusc.2010.11.049

[122] Ravanbakhsh H Karamzadeh V Bao G Mongeau L Juncker D Zhang YS. Emerging technologies in multi-material bioprinting. Advanced mater (Deerfield Beach Fla) (2021) 33(49):e2104730. doi: 10.1002/adma.202104730 PMC8971140 34596923

[123] Homan KA Kolesky DB Skylar-Scott MA Herrmann J Obuobi H Moisan A. Bioprinting of 3d convoluted renal proximal tubules on perfusable chips. Sci Rep (2016) 6:34845. doi: 10.1038/srep34845 27725720 PMC5057112

[124] Pourmasoumi P Moghaddam A Nemati Mahand S Heidari F Salehi Moghaddam Z Arjmand M. A review on the recent progress, opportunities, and challenges of 4d printing and bioprinting in regenerative medicine. J biomater Sci Polymer ed (2022) 34(1), 108–146. doi: 10.1080/09205063.2022.2110480 35924585

[125] Fu CY Tseng SY Yang SM Hsu L Liu CH Chang HY. A microfluidic chip with a U-shaped microstructure array for multicellular spheroid formation, culturing and analysis. Biofabrication (2014) 6(1):15009. doi: 10.1088/1758-5082/6/1/015009 24589876

[126] Nguyen NT Shaegh SA Kashaninejad N Phan DT. Design, fabrication and characterization of drug delivery systems based on Lab-on-a-Chip technology. Advanced Drug delivery Rev (2013) 65(11-12):1403–19. doi: 10.1016/j.addr.2013.05.008 23726943

[127] Wen Y Yang ST. The future of microfluidic assays in drug development. Expert Opin Drug Discovery (2008) 3(10):1237–53. doi: 10.1517/17460441.3.10.1237 23489080

[128] Vadivelu RK Kamble H Shiddiky MJA Nguyen NT. Microfluidic technology for the generation of cell spheroids and their applications. Micromachines (2017) 8(4):94. doi: 10.3390/mi8040094

[129] Shukla P Yeleswarapu S Heinrich MA Prakash J Pati F. Mimicking tumor microenvironment by 3d bioprinting: 3d cancer modeling. Biofabrication (2022) 14(3). doi: 10.1088/1758-5090/ac6d11 35512666

[130] Mazzaglia C Sheng Y Rodrigues LN Lei IM Shields JD Huang YYS. Deployable extrusion bioprinting of compartmental tumoroids with cancer associated fibroblasts for immune cell interactions. Biofabrication (2023) 15(2). doi: 10.1088/1758-5090/acb1db 36626838

[131] Grolman JM Zhang D Smith AM Moore JS Kilian KA. Rapid 3d extrusion of synthetic tumor microenvironments. Advanced mater (Deerfield Beach Fla) (2015) 27(37):5512–7. doi: 10.1002/adma.201501729 PMC4745120 26283579

